# A Rare Case of Severe Preeclampsia and HELLP (Hemolysis, Increased Liver Enzymes, Low Platelets) Syndrome With Complex Clinical Presentation

**DOI:** 10.7759/cureus.67127

**Published:** 2024-08-18

**Authors:** Vidya Gaikwad, Jay Patel, Suhas Gaikwad, Sneha Aramandla, Rushikesh Phutane

**Affiliations:** 1 Obstetrics and Gynaecology, Dr. D. Y. Patil Medical College, Hospital and Research Centre, Dr. D. Y. Patil Vidyapeeth (Deemed to be University), Pune, IND

**Keywords:** vancomycin-induced nephrotoxicity (vin), acute interstitial nephritis (ain), acute tubular injury (ati), acute kidney injury (aki), disseminated intravascular coagulation (dic), severe preeclampsia, gynaecology and obstetrics, hellp

## Abstract

Severe preeclampsia is a disorder of pregnancy, characterized by increased blood pressure (>140/90 mmHg) and proteinuria (≥ 300 mg/24 hours) at later than 20 weeks of gestation. Particularly in underdeveloped nations, severe preeclampsia and eclampsia have a significant negative impact on the health of expectant mothers, fetuses, and newborns. The HELLP (hemolysis, increased liver enzymes, low platelets) syndrome is thought to be a subset of preeclampsia, a group of hypertensive disorders of pregnancy that also includes eclampsia. Compared to preeclampsia alone, maternal and fetal problems are more severe in HELLP. There can be a diagnostic dilemma that arises when attempting to differentiate HELLP from its numerous imitators to determine the appropriate course of treatment.

Here, we present a rare case of a pregnant woman presenting with preeclampsia complicated by manifestations and investigations suggestive of HELLP syndrome with acute kidney injury (AKI), retinal detachment, and symptoms of DIC (disseminated intravascular coagulation), which can be grievous to the mother as well as the fetus.

## Introduction

Hypertensive disorders are associated with increased rates of maternal, fetal, and infant mortality and severe morbidity [1]. Complications of pregnancy-induced hypertensive disorders are more commonly encountered in low- and middle-income nations [2]. HELLP (hemolysis, increased liver enzymes, low platelets) is a triad of hemolysis with a microangiopathic blood smear, elevated liver enzymes, and a low platelet count defined in 1982 [3]. Adverse clinical outcomes, such as hepatic infarction, disseminated intravascular coagulation (DIC), renal failure, and pulmonary edema, are linked to HELLP syndrome [4]. Most often, HELLP syndrome occurs in the third trimester [5]. Severe preeclampsia/eclampsia is associated with 50,000-100,000 annual deaths globally [6]. Severe proteinuria, hypertension, central nervous system dysfunction symptoms, hepatocellular injury, thrombocytopenia, oliguria, pulmonary edema, cerebrovascular accident, and severe intrauterine growth restriction are characteristics of severe preeclampsia [7]. The wide differential of preeclampsia with HELLP and other symptoms, including thrombocytopenia, hepatitis, cholecystitis, acute fatty liver of pregnancy, and other microangiopathic hemolytic illnesses like thrombotic thrombocytopenic purpura (TTP) and atypical hemolytic-uremic syndrome (aHUS), might lead to misdiagnosis [5]. So clinicians need to make an accurate diagnosis of these conditions and prevent any adverse outcomes.

## Case presentation

**Figure 1:**
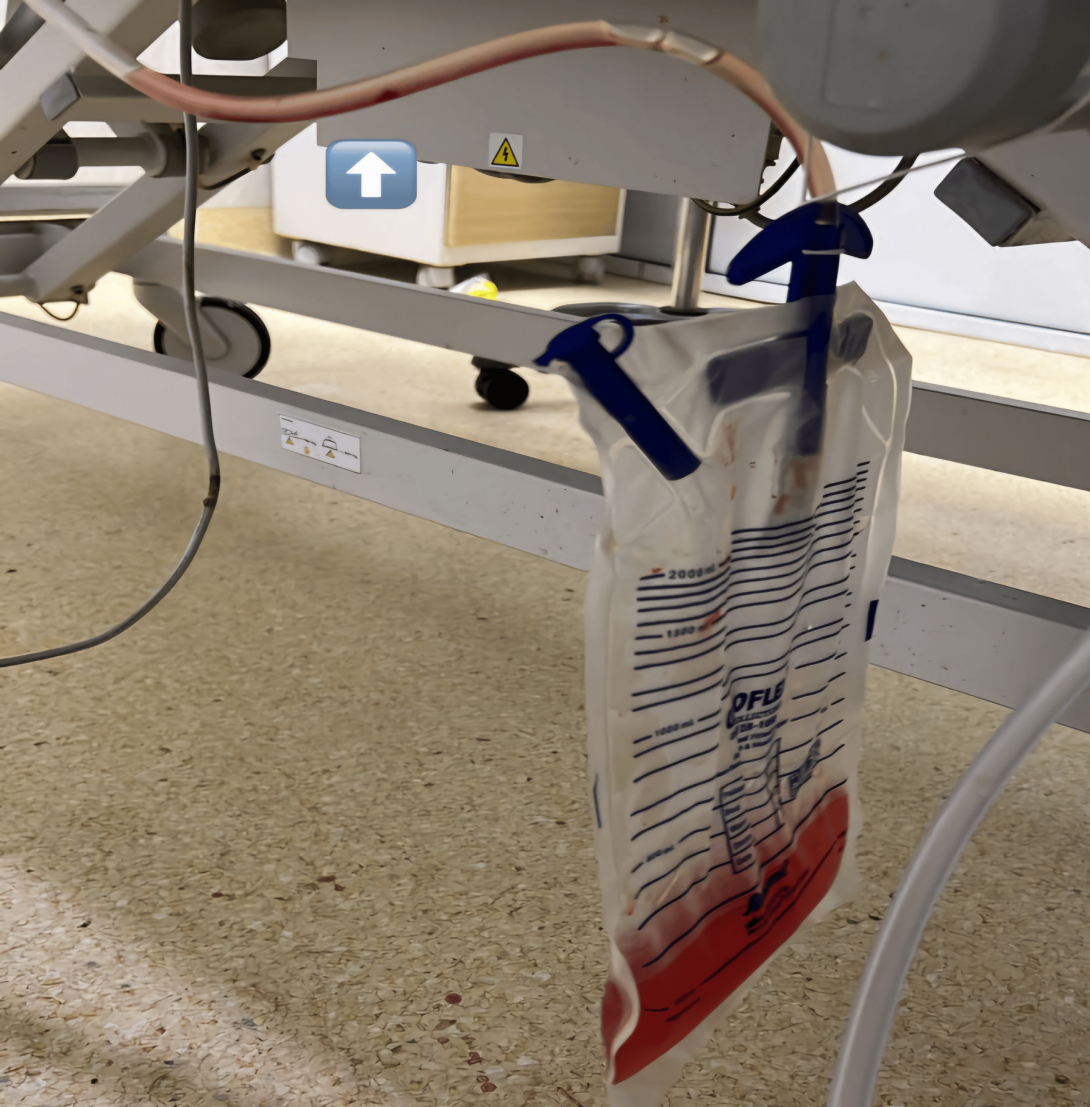
Hematuria

A 30-year-old, gravida 2, at 35 weeks gestation, with a previous full-term normal vaginal delivery, presented to the labor room of Dr. D.Y. Patil Medical College Hospital and Research Centre with complaints of epigastric pain for two days and bilateral pedal edema for one month before admission. She was in latent labor. She received an intravenous (IV) magnesium sulfate (MgSO_4_) loading dose of 4g (according to Pritchard's regimen), labetalol 100 mg tablet, and injection (Inj) betamethasone 12 mg before being referred here. The patient had an indwelling Foley catheter with hematuria on presentation (Figure 1).

On admission, blood pressure (BP) was 134/90 mmHg, with a pulse of 112 bpm and a urine albumin dipstick showing +3. On per-vaginal (PV) examination, the cervix was 2 cm dilated and 30% effaced. The systemic examination was normal. A diagnosis of severe preeclampsia in preterm labor was made. On admission, a complete hemogram, urine routine microscopy, liver function test, renal function test, and other routine investigations were performed (Tables 1, 2).

**Table 1:** Blood sample investigations HB: hemoglobin; TLC: total leukocyte count; SGOT: serum glutamic oxaloacetic transaminase; ALP: alkaline phosphatase; LDH: lactate dehydrogenase; PT/INR: prothrombin time/international normalized ratio; aPTT: activated partial thromboplastin time.

Parameter	Normal Range	14/04/2024 (1.1) (on admission)	14/04/2024 (1.2) (2 hours post-delivery)	15/04/2024 (1.3)	16/04/2024 (1.4)	17/04/2024 (1.5)	19/04/2024 (1.6)	29/04/2024 (1.7)
HB	12-15 g/dl	15	9.3	9.3	9.2	10.1	9.1	7.8
TLC	4000–11,000/μl	14,700	14,500	14,000	14,500	18,200	12,000	7,900
Platelets	1,50,000-4,00,000/μl	1,50,000	32000	36,000	34,000	56,000	76,000	2,10,000
Bilirubin T	0.22-1.20 mg/dl	1.52	4.53	3.68	1.43	0.98	1.42	
SGOT	8-43 U/Lt	119	309	679	140	68	47	
SGPT	7-45 U/Lt	65	637	284	112	67	39	
ALP	35-104 U/Lt	261	170	137	99	93	93	
LDH	81-234 U/Lt	457	-	2007	742	620	572	
Fibrinogen	238-498 mg/dl	337	-	228	235	230	261	
Urea	17-49 mg/dl	22	61	76	75	114	80	49
Creatinine	0.6-1.2 mg/dl	0.75	1.97	2.31	2.48	3.73	3.7	1.84
PT/INR	10.24-12.71 sec / <1.15	8.8	14.1	11.7	11.48	11.48	11.48	
aPTT	24.7-34.30 s	29.5	37.5	29.8	53.8	24.7	24.5	
D-Dimer	0-500 ng/ml		>10,000	7912	2014	1866	3714	

**Table 2:** Urine sample investigation RBC: red blood cells.

Urine Parameter	Normal Range	Patient value
Appearance	-	Very cloudy
Specific gravity	1.003-1.035	1.025
pH	4.6-8.0	8
Protein	Absent	2+
Glucose	Absent	1+
Acetone	Absent	Trace
Bile pigments	Absent	Absent
RBCs	0-2 / hpf	90-100
Pus cells	0-5 / hpf	3-4
Epithelial cells	0-5 / hpf	1-2

An electrocardiogram (ECG) showed a right bundle branch block. 

Cerviprime induction was done. The patient delivered vaginally approximately four hours later. The baby cried immediately after birth, with an Apgar score of 8/10. Postpartum blood pressure was 150/100 mm Hg. A tablet of labetalol (100 mg) was given. There was no evidence of postpartum hemorrhage. A bout of fresh hematuria was seen in the urobag immediately after delivery. Inj Tranexamic Acid 1 g intravenously and Inj Vit-K 10 mg were given. After receiving four packs of fresh frozen plasma (FFP), the patient was sent to the intensive care unit (ICU). She was managed by a multidisciplinary team approach (nephrologist, physician, urosurgeon, and obstetrician) with a clear management plan. Repeat investigations are shown in Table 1 (see columns 1.2 and 1.3). Based on the laboratory investigations, she was diagnosed with severe preeclampsia with HELLP syndrome with DIC and acute kidney injury (AKI). 

The nephrologist advised adequate intravenous fluids along with a single-donor platelet (SDP) transfusion. Ultrasonography (USG) showed blood clots in the renal cortex and bladder with moderate ascites and bilateral pleural effusion. The urology department advised a continuous saline wash of the bladder to displace and remove the clot with a three-way catheter. The 2D echo findings were normal. Inj Meropenam was started. The Inj Torsemide infusion, along with the Inj Lasix stat dose, was given as per the nephrology advice. The first dose of plasmapheresis was administered. The patient developed a blurring of vision two days after the delivery. A B-scan of the eyes was suggestive of left vitreous detachment, for which prednisolone eye drops were started by the ophthalmologist. There was a significant rise in abdominal girth with pedal edema, vulval edema, and anuria. The patient was given dialysis. A tablet of labetalol (100 mg) and nifedipine (10 mg) were given as antihypertensives. Magnesium sulfate dressing was done regularly for vulval edema. The nephrologist started intravenous steroid methylprednisolone (5 mg) BD. The patient started to have a urine output of 600 mL/day on the third day of delivery.

The patient was shifted out of the ICU to the ward after seven days of delivery. Symptoms were improving, vision was better, and laboratory values were found to be comparatively on the normal side (Table 1, column 1.6). The urinary catheter was removed after an examination by the urologist. Strict blood pressure and input/output monitoring were continued. Four FFPs, one PCV, one SDP, and 12 cryoprecipitates were given for a total duration of seven days, along with plasmapheresis and dialysis, for the management of this patient. The patient was discharged after 17 days of hospital stay. Regular follow-up was done in the ObGyn and Nephrology outpatient departments (OPDs).

## Discussion

It was discovered that an 8.7-fold increased risk of composite maternal complications was linked to severe preeclampsia [8]. Hepatic rupture, DIC, acute renal failure, cerebral hemorrhage, and pulmonary edema are the causes of maternal death [9, 10]. Chronic renal failure, cardiovascular illness, or cortical blindness are examples of long-term consequences [11].

The last sign of an insult that causes intravascular platelet aggregation and microvascular endothelial damage appears to be the HELLP syndrome [5]. Fibrinogen polymerizes into fibrin strands after being activated by thrombin. These fibrin strands create mesh-like barriers in small blood vessels, which leads to the death of red blood cells and the syndrome's hallmark microangiopathic hemolytic anemia. Low platelet counts are caused by platelets adhering to fibrin formations. Reduced hepatic perfusion and ischemia result from clot formation in the hepatic vasculature, which can cause an infarction and liver failure if left untreated [11]. Liver dysfunction and failure result in the elevated liver enzymes characteristic of HELLP syndrome [12]. It makes up between 0.2% and 0.6% of all pregnancies. Ten percent of cases of severe preeclampsia and almost 50% of cases of eclampsia are caused by this disorder [13].

The Tennessee Classification System established strict criteria for complete HELLP syndrome and the patient fulfilled these criteria: low platelets, defined as ≤100 x 109/L, raised values in liver function tests, defined as aspartate transaminase (AST) or alanine transaminase (ALT) more than or equal to twice the upper limit of the normal, and intravascular hemolysis, defined as elevated lactate dehydrogenase (LDH) (>600 U/L), total serum bilirubin, and/or an abnormal peripheral blood smear.

A majority of the time HELLP signs and symptoms appear between 28 and 36 weeks of gestation (70%) or within 48 hours after delivery (30%), and they usually subside in three to four days [14, 15]. This was similar in our patient as well, as her symptoms started at 35 weeks of pregnancy and within 24 hours of delivery. While it is perhaps occasionally recognized as a severe form of preeclampsia, some people with HELLP may not have a history of hypertension or proteinuria [16, 17]. AKI has been reported to occur in 7-60% of patients in the setting of HELLP syndrome alone [18, 19]. Sudden blindness brought on by involvement of the retina or occipital brain is one of the uncommon consequences of severe preeclampsia on the eye [20].

Stillbirths, iatrogenic prematurity and its aftereffects, and low or extremely low birth weight are examples of fetal/neonatal problems [21].

For preeclampsia/HELLP, there is no specific treatment available other than delivering the fetus immediately. Studies on the use of corticosteroids have yielded inconsistent findings. A Cochrane analysis revealed that there was virtually no difference between corticosteroids and placebo or no treatment in terms of the risk of maternal death, severe maternal morbidity, or perinatal or infant death [22]. To prevent a cerebrovascular event, it is advised to take additional precautions, such as using magnesium sulfate and blood pressure control using pregnancy-safe hypertension drugs, to stop the progression to eclampsia [23, 24].

## Conclusions

This was a rare presentation of a complex case of severe preeclampsia with HELLP, DIC, and AKI. The standard test findings aided in the diagnosis. A logical, sequential approach to diagnosis and management can prevent the costly consequences of a missed diagnosis and maternal and neonatal morbidity and mortality. Here, a multidisciplinary approach by obstetricians, physicians, nephrologists, and urologists with aggressive supportive care helped to resolve the end-organ involvement of severe preeclampsia completely.
